# Longitudinal effects of time since injury and age at injury on outcomes of people with spinal cord injury in Queensland, Australia

**DOI:** 10.1038/s41393-022-00824-8

**Published:** 2022-06-28

**Authors:** Melissa B. Kendall, Delena Amsters, Sarita Schuurs, David N. Borg, Kiley Pershouse, Pim Kuipers

**Affiliations:** 1 Transitional Rehabilitation Program, Queensland Spinal Cord Injuries Service, Metro South Health, Brisbane, QLD Australia; 2 Griffith University, Menzies Health Institute Queensland, The Hopkins Centre, Brisbane, QLD Australia; 3 Spinal Outreach Team, Queensland Spinal Cord Injuries Service, Metro South Health, Brisbane, QLD Australia

**Keywords:** Quality of life, Rehabilitation

## Abstract

**Study design:**

Longitudinal cohort study.

**Objectives:**

To investigate the longitudinal effects of time since injury and age at injury on outcomes of quality of life, physical function, secondary conditions and participation, in people with traumatic spinal cord injury (SCI).

**Setting:**

Community resident people with spinal cord injury in Queensland, Australia.

**Methods:**

A baseline sample of 270 people with SCI was recruited. Telephone surveys on measures of quality of life (WHOQOL-Bref), secondary conditions (Secondary Conditions Surveillance Instrument, subset), physical functioning (Functional Independence Measure motor subscale) and participation (Community Integration Measure) were conducted each year between 2004 and 2008, and again in 2018. Random-effect within-between models were used to determine the effect of time since injury and age at injury on each outcome variable. Inverse probability-of-censoring weights were used to correct for selection bias.

**Results:**

There was an effect of time since injury on secondary conditions, with a one-year change associated with 9% higher odds of having worse Secondary Conditions Surveillance Instrument scores (odds ratio = 1.09, 95% confidence interval = 1.02, 1.17; *p* = 0.006). We did not find any evidence of a time since injury effect on quality of life, physical function, or participation. Similarly, we did not find any evidence of an age at injury effect on any outcome variable.

**Conclusions:**

Secondary conditions may increase with longer time since injury among people with SCI, suggesting appropriate formal and informal supports are required to minimise the impact of these emerging health problems as individuals age.

## Introduction

Human function is described in the International Classification of Functioning Disability and Health (ICF) as a ‘dynamic interaction between a person’s health condition, environmental factors and personal factors’ [1]. Thus, a person with a persisting condition such as traumatic spinal cord injury (SCI) may experience a change in function over time as the intrinsic and extrinsic influencing factors are unlikely to remain constant. Several research groups have endeavoured to uncover patterns of change over time, to help predict and comprehensively plan for the needs of people with SCI as they age [2–5].

Researchers have highlighted that the interactions between physiological ageing and SCI are complex [3, 5]. Still, there are suggestions that people with SCI may age more rapidly both biologically and functionally [6]. Adding to the complexity is a broad trend suggesting that the age of SCI onset is also shifting, trending towards older age at the time of injury [7]. Some research suggests that people who experience SCI onset at an advanced age are more likely to experience a more rapid decline in function [8], possibly due to decreased capacity for physical adaptability when age related changes are already present [9]. Others suggest that age at onset may have a greater influence on participation than physical function or secondary conditions [10].

Equally, time since injury is an important factor to consider. Research suggests that quality of life for people with SCI remains comparatively stable over time [4, 10, 11], albeit at a level which is significantly lower than population norms [11]. Physical function however may display a more varied trajectory. Some research suggests that physical function remains quite stable or improves during the first 20 to 25 years following injury, followed by a decline in function during latter years [3, 4]. A study by Gerhart et al. [12] examining self-reported changes in physical function over time in a British cohort of 279 individuals, found that participants with cervical level injuries required increased care support at a younger age than those with lower-level injuries. In a retrospective study conducted with an Australian cohort of people with long duration SCI, 27% reported a decline in mobility and 26% reported a decrease in their ability to perform self-care over time [13]. This study also suggested that those with tetraplegia and those with neurologically incomplete injuries showed a more marked decrease in physical function compared with participants with paraplegia [13].

Secondary health conditions following SCI have rarely been investigated as an outcome per se and there is a need to track the course of these conditions over time and the implications for people’s experience of disability over the long term [14]. Studies have generally described the negative influence of secondary conditions on long-term outcomes for people with SCI [9, 15, 16]. The contributory impact of age on the severity and functional consequences of secondary conditions after SCI has also been noted [9, 15, 17]. Indeed, secondary conditions associated with SCI, such as musculoskeletal pain, pressure injuries, spasticity and bowel management issues have been associated with a lower quality of life [15].

While a subtle decline in (objective) participation seems to be a feature of ageing with SCI, the subjective experience is more mixed [18]. Previous research has revealed an increase in participation with increasing time since injury but not when adjusted for impairment, income, and social situation [4]. It is likely that participation will increase with time, as knowledge, skills and networks of support are developed. However, declining health and increasing secondary conditions [4] compounded by the presence of barriers to community access [19] may be compromising factors.

To ensure appropriate timing and availability of services and supports across the lifespan for people with traumatic SCI, accurate information regarding how outcomes change over time and in relation to age at onset is needed [20]. Therefore, the purpose of the current study was to investigate the longitudinal effects of time since injury and age at injury on change in outcomes of quality of life, physical function, secondary conditions, and participation.

## Methods

### Design

The study followed a longitudinal cohort design, with people with SCI followed over a 15-year period. Building on an initial 5-year data collection between 2004 and 2008 [4], participants were contacted in 2018, 10 years after the initial data collection period. Participants were invited to take part in a telephone survey, focusing on measures of quality of life, secondary conditions, physical functioning, and participation. These survey tools are described below. The same research assistant (physiotherapist) who collected most of the original survey data between 2004 and 2008 was responsible for data collection in 2018. The 2018 surveys were completed over a period of 35 weeks, with each telephone call taking between 10 and 35 min to complete. The protocol for this study was approved by the Metro South Health Human Research Ethics Committee (HREC/17/QPAH/819). As this was a cohort study, the STROBE guidelines for cohort studies were followed for reporting purposes.

### Setting

The study was conducted with community resident persons with traumatic SCI in Queensland, Australia.

### Participants

A longitudinal survey of people with traumatic SCI was conducted from 2004 to 2008 with data collected annually. The sample for the preliminary study has been reported in previous articles [4, 11]. It used a wave panel design, with 270 participants with SCI randomly selected from archival records of the Queensland Spinal Cord Injuries Service (QSCIS). In the initial study, participants were recruited in time since injury strata. This approach ensured a representative distribution of participants with a time since injury, ranging from 0 years to more than 25 years. In the current study, we treated time since injury as a continuous variable, rather than according to the six strata used in the original investigation [4, 11].

Participant demographic and injury-related characteristics are reported in Table 1. Over the course of the study, the total attrition rate was 42.6% (115/270) with 14.4% (39/270) due to death. The attrition rate during the period of yearly data collection between 2004 and 2008 was 13.3% (*n* = 36/270; 2.6% due to death). Of the 234 individuals who completed the surveys in 2008, 222 agreed to future contact from the researchers with 155 completing the final survey in 2018. The attrition rate between 2008 and 2018 was 29.3% (79/270) with 11.8% (32/270) deceased. Of those deceased, the majority (28/32) had a time since injury of 15 years or more.

**Table 1:** Demographic and injury-related characteristics, and living situation and paid employment status.

Variable	*n* = 270
Age (years)	43 (35–50)
Age at time of injury (years)	24 (20–32)
Time since injury (years)	15 (8–23)
Gender	
Male	218 (81%)
Female	52 (19%)
Level of injury	
Paraplegia	126 (47%)
Tetraplegia	144 (53%)
Completeness of injury	
Incomplete	145 (54%)
Complete	125 (46%)
Place of residence	
Metropolitan	139 (51%)
Non-Metropolitan	131 (49%)
Marital status	
Married or defacto	137 (51%)
Not living as married	133 (49%)
Living situation	
Living with others	209 (77%)
Living alone	61 (23%)
Employment status	
In paid workforce	112 (42%)
Not in paid workforce	158 (58%)

Continuous variables are summarised as the median (interquartile range) and categorical variables as count (percentage).

#### Measures

Demographic details were drawn from self-report within the survey and injury characteristics were drawn from archival medical records. Primary impairments were measured according to neurological level and completeness of injury, based on the American Spinal Injury Association (ASIA) classification system. Functionally complete injuries were those classified as ASIA A, B or C. Functionally incomplete injuries were those with neurological sparing (ASIA D) such that ambulation is typically possible.

Quality of life was measured using the World Health Organisation’s Quality of Life Instrument (WHOQOL)-Bref [21]. This self-report measure assesses perceived quality of life over the preceding 2 weeks on 26 items and has been previously used in SCI populations. For the current study, the scale was used in two ways; (a) using the two global items ‘how would you rate your quality of life’ and ‘how satisfied are you with your health’ and (b) across the four domain subscales (Physical, Psychological, Social and Environment).

The ICF outlines three components of human function––body structure/function, activity, and participation. Measures were chosen to reveal changes across these three components. Body structure/function was measured using a subset of the self-report Secondary Conditions Surveillance Instrument (SCSI) [22]. The full SCSI (range, 0–120) measures the severity of secondary conditions or problems experienced over the past year across 40 items on a 4-point scale from 0 (no problem) to 3 (significant problem). The 15-item subset (range, 0–45) selected for this study included those items that were identified as prevalent and relevant to people with SCI. This included items related to pressure sores, posture, bladder and bowel function, dysreflexia and fatigue as well as cardiovascular, circulatory and respiratory problems. The SCSI pain item was not included as a question as a pain item exists within the WHOQOL-Bref. Higher scores on the SCSI indicate more significant problems associated with secondary conditions.

The motor subscale of the Functional Independence Measure (mFIM) was used to measure activity [23]. The mFIM (range 7–91) rates functional independence on a scale of one to seven across 13 items and has proved valid and reliable when administered by telephone to people with SCI [24]. Higher scores indicate higher functional independence.

Participation was measured with the Community Integration Measure (CIM) [25], a psychometrically sound measure of community integration when used in the SCI population [26]. The CIM (range 10–50) assesses perception of connection with community on a 5-point scale from 1 (always disagree) to 5 (always agree) across 10 items. Higher scores indicate greater community integration.

### Analysis

All analyses were performed in R (version 4.0.3). Our primary interest was to investigate the effects of time since injury and age at injury on outcomes of quality of life, secondary conditions, physical functioning, and participation. All models included *year*, *injury level*, *completeness*, *time since injury*, *age at injury*, and whether individuals lived alone (*living status*) as fixed effects. Because year and time since injury were correlated, within-between estimation was used to model time since injury, separating inter-individual variation (i.e. between-subject effects) and intra-individual change (i.e. within-subject effects) across the six measurement points (i.e. 2004–2008, and 2018)—with the intra-individual change the effect of interest [27]. See Appendix 2.2 in Reinhardt et al. [28] for an explanation of between-within estimation. All models included inverse probability-of-censoring weights as a fixed effect, to correct for potential bias induced by loss to follow-up [29]. Inverse probability-of-censoring weights are based on an estimate of how loss to follow-up depends on observed baseline and time-updated participant characteristics. Weights were calculated using the R package *mets* [30], with injury level, completeness, gender, age at injury, and time since injury (separated for within-subject and between-subject effect) included in the model. All models included a random intercept and slope (year) for each participant in the study.

Ordinal regression was used to model ratings of WHOQOL-Bref QOL and Health Satisfaction. Stan [31] with the *brms* interface [32] was used to fit a cumulative model and an adjacent category model [33] with the best fitting model selected based on the smallest Leave-One-Out Information Criterion. Weakly informative prior distributions were set for the regression coefficients and variance parameters [34]. A Gaussian (mean 0, standard deviation 1) prior distribution was used for the regression coefficients, and a *t*-distribution (df 3, mean 0, scale 2.5) prior for the standard deviation of the random effects. In this setting, the prior distribution on the fixed effects has a 95% interval of −1.95 and 1.95—equivalent to an odds ratio of 0.14 and 7.03, respectively. Posterior estimates were generated using Markov chain Monte Carlo methods (16,000 iterations, with a 50% burn-in) and are reported as the mean and 95% credible interval.

WHOQOL-Bref Physical, Psychological, Social and Environment data were modelled with a Gaussian response distribution, and SCSI with a Poisson response distribution. These models were fit using the R package *panelr* [35]. mFIM and CIM data were modelled with a beta response distribution, which assumes a continuous, interval-level response variable, with known bounds [36]. The beta distribution is highly flexible and can accommodate heteroscedasticity and skewed errors [36]. mFIM and CIM scores were rescaled to lie within the (0, 1) interval using the equation: *y’* = (*y* − *a*)/(*b* − *a*), where ‘*a*’ is the minimum possible questionnaire score, ‘*b*’ the maximum possible score, and ‘*y*’ the observed score. Because the beta distribution cannot take responses that are exactly one, ones were shifted off the boundary by or subtracting 0.00001 [36]. This has no impact on scores when back transformed to the original scale. Beta regression models were fit using the *mgcv* package [37].

Data are reported as the mean and 95% confidence interval (CI), unless otherwise stated. The α level for all tests was set at 5%. The R code used to generate the study findings can be found at https://github.com/SciBorgo/longterm-function-sci-qld.

## Results

The main finding of the study was that a longer time since injury was associated with more secondary conditions, with a 1-year change associated with 9% higher odds of having worse SCSI scores. We did not find any evidence that time since injury or age at injury had an effect on any of the other studied outcome variables. The study results are described in further detail below.

**Fig. 1:**
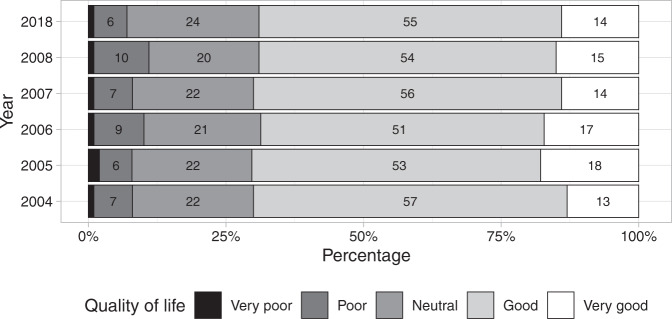
Ratings of Global Quality of Life on the World Health Organisation’s Quality of Life Instrument (WHOQoL-Bref) from 2004 to 2008 and in 2018. The labels within each horizonal bar are percentage responses. Labels for ‘Very poor’ are not shown. The response sample size for each year was: *n* = 270 (in 2004), *n* = 261 (2005), *n* = 247 (2006), *n* = 240 (2007), *n* = 234 (2008) and *n* = 155 (2018).

**Fig. 2:**
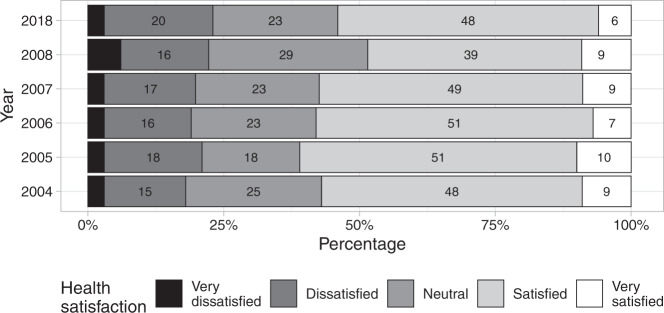
Ratings of Health Satisfaction on the World Health Organisation’s Quality of Life Instrument (WHOQoL-Bref) from 2004 to 2008 and in 2018. The labels within each horizonal bar are percentage responses. Labels for ‘Very poor’ are not shown. The response sample size for each year was: *n* = 270 (in 2004), *n* = 261 (2005), *n* = 247 (2006), *n* = 240 (2007), *n* = 234 (2008) and *n* = 155 (2018).

WHOQOL-Bref global QOL ratings are shown in Fig. 1; and Health Satisfaction ratings in Fig. 2. The cumulative ordinal model was a better fit compared to the adjacent category model for global QOL (Leave-One-Out Information Criterion = 2587 versus 2631) and Health Satisfaction (Leave-One-Out Information Criterion = 2906 versus 2944). There was no evidence of time since injury or age at injury effects on global QOL or Health Satisfaction (Supplementary 1).

**Fig. 3:**
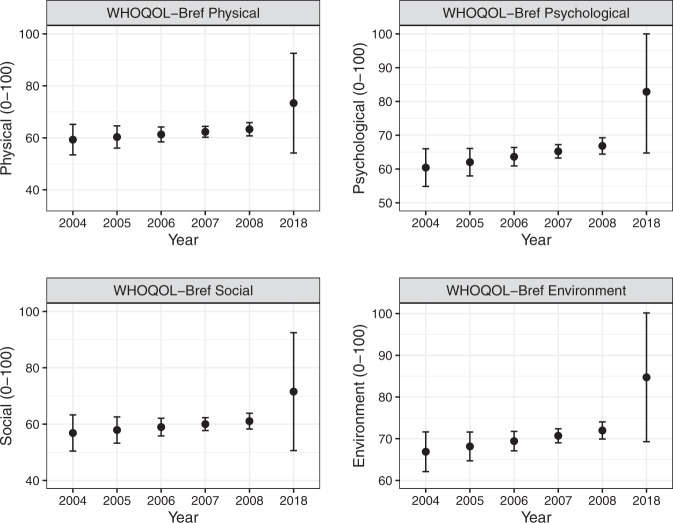
Physical, Psychological, Social and Environment subscale scores on the World Health Organisation’s Quality of Life Instrument (WHOQoL-Bref) from 2004 to 2008 and in 2018. Data are reported as the mean and 95% confidence interval.

Figure 3 shows WHOQOL-Bref scores on the Physical, Psychological, Social and Environment QOL subscales. There was no evidence of time since injury or age at injury effects on Physical, Psychological, Social and Environment QOL scores (Supplementary 2). There was, however, evidence that participants living with others had higher Social QOL scores compared to those who lived alone (*β* = 7.2, 95% CI = 4.4, 10.1, *p* < .001). It was unclear whether there was an effect of year on Psychological QOL scores (*β* = 1.60, 95% CI = −0.05, 3.26, *p* = 0.058).

**Fig. 4:**
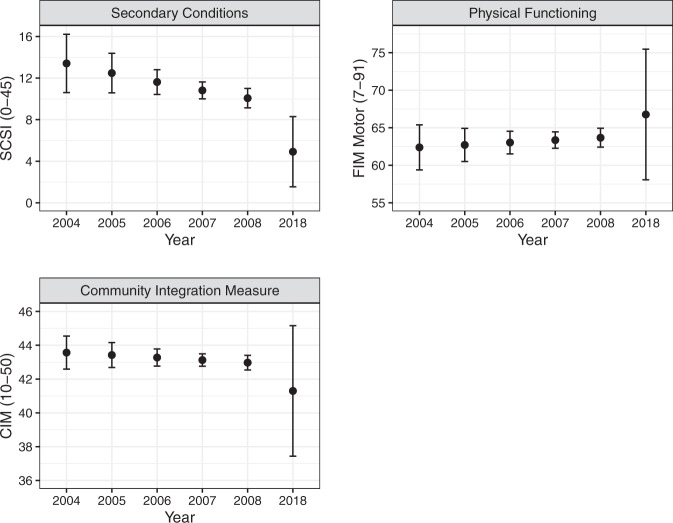
Secondary Conditions Surveillance Instrument (SCSI), Functional Independence Measure (FIM) Motor subscale, and Community Integration Measure (CIM) scores from 2004 to 2008 and in 2018. Data are reported as the mean and 95% confidence interval.

Secondary Conditions Surveillance Instrument scores are shown in Fig. 4. A longer time since injury (within-subject effect) was associated with more secondary conditions (*β* logit = 0.09, 95% CI = 0.3, 1.5, *p* = 0.006), with a 1-year change associated with 9% higher odds of worse (i.e. higher) SCSI scores (odds ratio [OR] = 1.09, 95% CI = 1.02, 1.17). There was also an effect of year on SCSI scores (*β* logit = −0.07, 95% CI = −0.13, −0.01, *p* = 0.025), with each year after 2004 associated with a 7% reduction in SCSI scores (OR = 0.93, 95% CI = 0.88, 0.99). We also found evidence that participants with an incomplete injury had fewer secondary conditions compared to those with a complete injury (mean difference = −2.0, 95% CI = −3.6, −0.4, *p* = 0.011). Analysis was not conducted at the item level. However, descriptively the highest aggregate impact on life (as worded in the scale itself) was noted for items related to fatigue, physical fitness, spasm, bladder and bowel problems. Therefore, these were the items that scored highest.

Figure 4 shows mFIM scores. There was no evidence of time since injury or age at injury effects on physical function. Participants with tetraplegia had lower physical function compared to those with paraplegia (mean difference = −15, 95% CI = −17, −13, *p* < 0.001). Participants with an incomplete injury had higher physical function than those with a complete injury (mean difference = 20, 95% CI = 18, 22, *p* < 0.001). Participants who lived with others also had higher physical function than those who lived alone (mean difference = 2.2, 95% CI = 0.1, 4.3, *p* = 0.041). CIM scores are displayed in Fig. 4. There was no evidence of time since injury or age at injury effects on participation as measured by the CIM (Supplementary 2).

## Discussion

This study aimed to examine the effects of time since injury and age at injury on outcomes of quality of life, physical function, secondary conditions, and participation. We found evidence that a longer time since injury was associated with more problems related to secondary conditions (OR = 1.09, 95% CI = 1.02, 1.17). We did not find any evidence of a time since injury effect on quality of life, physical function or participation, and no evidence of an age at injury effect on any outcome variable. Appropriate formal and informal supports are required to minimise the impact of these emerging health problems as individuals age with their injury.

While these findings suggest greater health problems arising from a SCI over time since injury, there did not appear to be parallel changes in quality of life, physical function, and participation. Existing literature suggests that the increased experience of secondary conditions impacts other outcomes including quality of life and participation [9, 15, 16, 19]. This was not evident in our findings. While the current study supports previous work noting little change in quality of life [11, 38] and participation [18] over time since injury, changes in physical function, particularly for those with higher level injuries, which has been shown in previous studies [3, 4, 12], were not evident.

Our findings are encouraging and may reflect the Australian service context for people with SCI. In Queensland, where the study was conducted, a statewide all-of-life continuity of care service model exists for people with SCI. This is supported by a national public health system. Collectively, these service models provide a unique combination of formal supports for individuals with SCI across the lifespan. Improvements in service models and delivery between 2004 and 2018 could partly explain why we found an effect of year on secondary conditions, with each year after 2004 associated with a 7% reduction in SCSI scores. Given the recent introduction of a National Disability Insurance Scheme (NDIS) providing access to formal supports and funding, the impact of secondary health conditions experienced with aging may be further diminished [38]. Future research is needed to examine these outcomes through comparison with population norms and within the context of the NDIS. Ultimately, parity in quality of life and participation, regardless of injury, would be the most desirable goal.

We did not identify any association between age at injury onset and any of the studied outcomes. This contradicts previous research [6, 8–10]. It is possible that the measures used in the current study were not sufficiently sensitive to detect any changes attributed to age at injury onset. There was also a relatively narrow interquartile range for age at injury in this cohort which may be explanatory. However, we did observe expected differences, such as higher physical function among those with paraplegia and incomplete injuries, which could indicate that the included measures were appropriately sensitive.

The current study found that living with at least one other person was associated with better social QOL and higher physical function. It is unclear whether individuals were living with others because they have better social QOL or physical function, or whether these outcomes were improved because participants were living with others. Regardless of the causal mechanisms, the social support available through informal supports may be particularly important for both social and physical function, a finding consistent with existing literature [39]. When assessing individuals for formal supports, it may be necessary to consider the living situations of people with SCI as they age and adjust supports accordingly.

### Limitations and future research

Our results may not be generalisable to all people with SCI. While accounted for in the data analysis, there was an attrition rate over the study duration of 42.6%. This highlights the challenges of conducting longitudinal research in this area. Study attrition, and the use of inverse probability-of-censoring weights, explains the large uncertainty on estimates in 2018 relative to the years 2004–2008. This uncertainty is reflected by much wider confidence intervals on all outcome measures presented in Figs. 3,  4. The large uncertainty on point estimates in 2018 likely explains why we did not find an effect of year on most outcomes (other than secondary conditions), despite seemingly lower or higher mean values in 2018 compared to the years 2004–2008 (e.g. community integration measure, Fig. 4). The current study was limited in the range of predictive/risk factors considered. It is feasible that other factors not taken into consideration may have influenced the findings over the 15-year-data collection period. Further research that explores the evolution of specific secondary conditions would be useful in targeting resources.

The current study provides limited insight into the individual experience of changes in quality of life, physical function, secondary conditions, and participation as it relates to time since injury and age at onset for people with SCI. Greater attention to qualitative investigations may provide greater understanding of people’s realities, their experiences, and the meanings they attribute to these issues in their lives.

## Conclusion

We found evidence that secondary conditions associated with SCI increased with a longer time since injury. However, the increase in secondary conditions did not appear to affect quality of life, physical function, or participation. The formal assistance provided by an appropriate and resourced health system combined with the informal support of living with others may be particularly beneficial for people with SCI as they age. Future research exploring secondary conditions for people with SCI as they live longer with their injury is warranted.

## Supplementary information

Supplement 1

Supplement 2

## Data Availability

The datasets generated and analysed during the current study are available from the corresponding author on reasonable request. The R code used to generate the study findings can be found at https://github.com/SciBorgo/longterm-function-sci-qld.
