# Supplementary material for: Longitudinal effects of time since injury and age at injury on outcomes of people with spinal cord injury in Queensland, Australia
Source: Spinal Cord. 2022 Jun 28;60(12):1087–93. doi: 10.1038/s41393-022-00824-8 (PMC9712094; doi:10.1038/s41393-022-00824-8)
Supplement: Supplementary file 1 — Supplement 1 [file 41393_2022_824_MOESM1_ESM.docx]

**Supplement 1.** Parameter estimates of fixed effects on the logit scale, from the WHOQOL-Bref Quality of Life and Health Satisfaction models.

| Parameter | Estimate | SD | Lower 95% CrI | Upper 95% CrI |
| --- | --- | --- | --- | --- |
| *WHOQOL-Bref Quality of Life* |  |  |  |  |
| Year | 0.17 | 0.13 | –0.07 | 0.42 |
| Level—tetraplegia | 0.44 | 0.30 | –0.17 | 1.03 |
| Completeness—incomplete | –0.15 | 0.30 | –0.75 | 0.44 |
| Age at injury | –0.03 | 0.02 | –0.06 | 0.01 |
| Inverse probability of censoring weight | 0.28 | 0.20 | –0.12 | 0.68 |
| Time since injury—within | –0.19 | 0.13 | –0.43 | 0.07 |
| Time since injury—between | –0.01 | 0.02 | –0.04 | 0.02 |
| Living status | 0.36 | 0.23 | –0.08 | 0.83 |
| *WHOQOL-Bref Health Satisfaction* |  |  |  |  |
| Year | –0.05 | 0.13 | –0.30 | 0.19 |
| Level—tetraplegia | 0.41 | 0.30 | –0.19 | 1.00 |
| Completeness—incomplete | 0.07 | 0.30 | –0.51 | 0.67 |
| Age at injury | –0.01 | 0.02 | –0.04 | 0.03 |
| Inverse probability of censoring weight | 0.33 | 0.20 | –0.05 | 0.71 |
| Time since injury—within | 0.02 | 0.13 | –0.23 | 0.27 |
| Time since injury—between | –0.002 | 0.016 | –0.034 | 0.029 |
| Living status | 0.14 | 0.22 | –0.29 | 0.59 |

*Note*. Intercept not shown. CrI = Credible interval, SD = Standard deviation.
