# Supplementary material for: Longitudinal effects of time since injury and age at injury on outcomes of people with spinal cord injury in Queensland, Australia
Source: Spinal Cord. 2022 Jun 28;60(12):1087–93. doi: 10.1038/s41393-022-00824-8 (PMC9712094; doi:10.1038/s41393-022-00824-8)
Supplement: Supplementary file 2 — Supplement 2 [file 41393_2022_824_MOESM2_ESM.docx]

**Supplement 2.** Parameter estimates of fixed effects from WHOQOL-Bref Physical, Psychological, Social and Environment models, and Secondary Conditions Surveillance Instrument, Functional Independence Measure Motor subscale (logit scale) and Community Integration Measure (logit scale) models.

| Parameter | Estimate | Standard error | Lower 95% CI | Upper 95% CI | *P*-value |
| --- | --- | --- | --- | --- | --- |
| *WHOQOL-Bref Physical* |  |  |  |  |  |
| Year | 1.00 | 0.89 | –0.74 | 2.75 | .26 |
| Level—tetraplegia | –2.29 | 2.10 | –6.41 | 1.83 | .28 |
| Completeness—incomplete | 2.67 | 2.09 | –1.43 | 6.77 | .20 |
| Age at injury | –0.15 | 0.12 | –0.37 | 0.08 | .20 |
| Inverse probability of censoring weight | –0.06 | 0.86 | –1.74 | 1.62 | .94 |
| Time since injury—within | –1.21 | 0.91 | –2.99 | 0.56 | .18 |
| Time since injury—between | –0.13 | 0.11 | –0.34 | 0.09 | .25 |
| Living status | –0.81 | 1.11 | –2.99 | 1.37 | .47 |
| *WHOQOL-Bref Psychological* |  |  |  |  |  |
| Year | 1.60 | 0.84 | –0.05 | 3.26 | .058 |
| Level—tetraplegia | 2.95 | 2.00 | –0.98 | 6.87 | .14 |
| Completeness—incomplete | 0.51 | 1.99 | –3.39 | 4.41 | .80 |
| Age at injury | –0.05 | 0.11 | –0.27 | 0.16 | .62 |
| Inverse probability of censoring weight | 1.04 | 0.79 | –0.52 | 2.59 | .19 |
| Time since injury—within | –1.52 | 0.86 | –3.20 | 0.16 | .077 |
| Time since injury—between | 0.08 | 0.10 | –0.13 | 0.28 | .47 |
| Living status | 1.43 | 1.07 | –0.67 | 3.52 | .18 |
| *WHOQOL-Bref Social* |  |  |  |  |  |
| Year | 1.05 | 0.97 | –0.85 | 2.95 | .28 |
| Level—tetraplegia | 0.34 | 2.33 | –4.22 | 4.91 | .88 |
| Completeness—incomplete | 0.91 | 2.32 | –3.62 | 5.45 | .69 |
| Age at injury | –0.09 | 0.13 | –0.34 | 0.16 | .48 |
| Inverse probability of censoring weight | 0.31 | 1.17 | –1.99 | 2.61 | .79 |
| Time since injury—within | –1.05 | 0.98 | –2.97 | 0.88 | .29 |
| Time since injury—between | 0.05 | 0.12 | –0.19 | 0.29 | .66 |
| **Living status** | **7.23** | **1.45** | **4.39** | **10.08** | **< .001** |
| *WHOQOL-Bref Environment* |  |  |  |  |  |
| Year | 1.27 | 0.72 | –0.13 | 2.68 | .077 |
| Level—tetraplegia | 0.41 | 1.69 | –2.91 | 3.73 | .81 |
| Completeness—incomplete | 1.64 | 1.68 | –1.66 | 4.94 | .33 |
| Age at injury | 0.13 | 0.09 | –0.06 | 0.31 | .18 |
| Inverse probability of censoring weight | –0.58 | 0.75 | –2.05 | 0.89 | .44 |
| Time since injury—within | –1.07 | 0.73 | –2.51 | 0.36 | .14 |
| Time since injury—between | 0.08 | 0.09 | –0.09 | 0.25 | .37 |
| Living status | 0.17 | 0.97 | –1.74 | 2.08 | .86 |
| *Secondary Conditions Surveillance Instrument (logit scale)* |  |  |  |  |  |
| **Year** | **–0.07** | **0.03** | **–0.13** | **–0.01** | **.025** |
| Level—tetraplegia | 0.11 | 0.08 | –0.04 | 0.26 | .15 |
| **Completeness—incomplete** | **–0.19** | **0.08** | **–0.34** | **–0.04** | **.011** |
| Age at injury | 0.005 | 0.004 | –0.003 | 0.013 | .26 |
| Inverse probability of censoring weight | 0.03 | 0.03 | –0.02 | 0.09 | .22 |
| **Time since injury—within** | **0.09** | **0.03** | **0.03** | **0.15** | **.006** |
| **Time since injury—between** | **0.01** | **0.00** | **0.00** | **0.02** | **.003** |
| Living status | 0.02 | 0.04 | –0.05 | 0.10 | .54 |
| *Functional Independence Measure Motor subscale (logit scale)* |  |  |  |  |  |
| Year | 0.02 | 0.03 | –0.03 | 0.07 | .46 |
| **Level—tetraplegia** | **–0.89** | **0.06** | **–1.00** | **–0.77** | **< .001** |
| **Completeness—incomplete** | **1.17** | **0.06** | **1.06** | **1.29** | **< .001** |
| Age at injury | 0.003 | 0.003 | –0.003 | 0.010 | .32 |
| Inverse probability of censoring weight | –0.04 | 0.10 | –0.24 | 0.15 | .65 |
| Time since injury—within | –0.02 | 0.03 | –0.08 | 0.03 | .37 |
| **Time since injury—between** | **–0.0064** | **0.0031** | **–0.0126** | **–0.0003** | **.04** |
| **Living status** | **0.13** | **0.06** | **0.01** | **0.26** | **.039** |
| *Community Integration Measure (logit scale)* |  |  |  |  |  |
| Year | –0.03 | 0.03 | –0.08 | 0.03 | .32 |
| Level—tetraplegia | –0.09 | 0.06 | –0.21 | 0.03 | .14 |
| Completeness—incomplete | –0.08 | 0.06 | –0.20 | 0.04 | .20 |
| Age at injury | –0.001 | 0.003 | –0.007 | 0.006 | .80 |
| Inverse probability of censoring weight | 0.01 | 0.10 | –0.18 | 0.21 | .91 |
| Time since injury—within | 0.03 | 0.03 | –0.02 | 0.09 | .23 |
| **Time since injury—between** | **0.007** | **0.003** | **0.001** | **0.014** | **.022** |
| Living status | –0.02 | 0.07 | –0.15 | 0.11 | .75 |

*Note*. Intercept not shown. CI = Confidence interval, SE = Standard error. Significant effects are shown in boldface.
